# Mast Cells: When the Best Defense Is an Attack?

**DOI:** 10.3390/ijms23073570

**Published:** 2022-03-25

**Authors:** Margarita Martin

**Affiliations:** 1 Biochemistry Unit, Biomedicine Department, Faculty of Medicine, University of Barcelona, 08036 Barcelona, Spain; martin_andorra@ub.edu; 2 Clinical and Experimental Respiratory Immunoallergy (IRCE), Institut d’Investigacions Biomèdiques August Pi i Sunyer (IDIBAPS), 08036 Barcelona, Spain; 3 ARADyAL (Asthma, Drug Adverse Reactions and Allergy) Research Network, 28029 Madrid, Spain

The main goal of this Special Issue was to highlight the recent advances made on the role of mast cells (MCs) in host defense and pathology. MCs are recognized as crucial initiators and regulators of both innate and adaptive immune responses against pathogens. MCs are resident tissue cells found throughout the body, mainly in association with blood vessels and nerves, and are preferentially observed at mucosal surfaces and the skin. This strategic location allows them to be sentinel cells, acting at the early stages of infection and promoting the recruitment of effector cells to sites of infection. MCs have been implicated in the host response to venoms, bacteria, parasites, and viruses. Multiple virus-associated stimuli can induce the production of type I and III IFNs, chemokines, inflammatory cytokines, as well as factors involved in tissue remodeling such as VEGF. In this issue, Darzianiazizi et al. [1] show that bone-marrow-derived MCs produced pro-inflammatory cytokines in response to recombinant vesicular stomatitis virus and that type I IFN receptor (IFNAR) signaling was required to downregulate these responses and protect the cells from dying from viral infection. This suggests that IFNAR signaling plays a central role in the antiviral cytokine responses of MCs. Upon MC stimulation, a large number of biologically active compounds are released, which afford a vast modulatory potential. Considerable attention has been given to the role of MCs in regulating T-cell function, either directly or indirectly, through actions on dendritic cells (DCs). In this issue, Taborska et al. [2] show that differentially stimulated MCs could be novel and highly potent cellular adjuvants for the maturation of DCs, and this ability opens a new avenue to use activated MCs for adoptive cellular immunotherapy. This procedure requires the maturation of ex vivo-produced immature DCs to mount an immune response against the antigen-expressing cells. Despite their beneficial role in host defense, MCs are better known for their detrimental contribution in several pathologies. Mastocytosis is a primary MC disorder where a high percentage of MCs carry the *KIT* D816V mutation, which makes MCs susceptible to proliferation and sensitive to degranulation. In this issue, Li [3] reviews the pathogenesis of systemic mastocytosis (SM), providing the updated view that coronavirus disease 2019 (COVID-19) does not impact MC activation symptoms in SM patients. The author discusses novel therapies such as the angiokinase inhibitor nintedanib (which targets VEGFR, PDGFR, and FGFR) and the use of inducible pluripotent stem cells (iPSCs) from patients as a model to tailor the disease and to improve the understanding of its pathogenesis. MCs are well known as the main effector cells in allergic inflammation and asthma (considered secondary MC disorders). In this issue, Berlin et al. [4] provide evidence that MC release of proteases (chymase and tryptase) induces morphological and functional alterations in bronchial epithelial cells, suggesting a critical role in airway epithelial remodeling and disruption of epithelial function. On the other hand, intraepithelial MC mediators disturb intestinal epithelial barrier function and are related to gastrointestinal disorders, such as inflammatory bowel disease, celiac disease, and irritable bowel syndrome. Thus, inhibitors of intestinal MC activity can be therapeutically used in these pathological conditions. In this Special Issue, Bilotta et al. [5] point to resveratrol, a natural inhibitor of human intestinal MC activation, as an anti-inflammatory nutraceutical in the treatment of MC-associated diseases. Moreover, Vera et al. [6] highlight lactones (dehydroleucodine, xanthatin, and 3-benzyloxymethyl-5H-furan-2-one) as valuable tools for designing novel therapeutic agents for digestive disorders since they show a cytoprotective effect on gastric mucosal lesions induced by inappropriate MC activation.

Precise knowledge about the mechanism underlying inappropriate MC activation will provide us with tools for diagnostics, prognosis, as well as treatment in MC-derived disorders. In this Special Issue, Garcia-Garcia et al. [7] review the anti- or pro-inflammatory activity of adenosine consistent with cell and tissue receptor expression (A1, A2a, A2b, and A3). Although the dual role of anti- and pro-inflammatory has been reported for A1, A2b, and A3 receptors, consistent anti-inflammatory action is found for the A2a receptor. Unfortunately, unwanted cardiovascular effects have been reported when using an A2a agonist as a treatment for lung diseases, and further research is required. The dysregulated expression of these receptors may lead to a pro-inflammatory phenotype and elicit an increase in the allergic response. Interestingly, higher expression of A3 has been found in food anaphylaxis patients. Severe allergic responses can lead to anaphylaxis, which may be a life-threatening condition if untreated. Guo et al. [8] review and highlight the role of transcription factors in anaphylaxis and related host genetic factors or mutations that underlie the molecular basis of this systemic hypersensitivity reaction. It is worth mentioning a GATA 3 expressing T-cell population that secretes IL13 defines the T follicular helper cell, Tfh13, which is required for the production of high- but not low-affinity IgE. Its presence may lead to anaphylactic IgE responses to allergens and can act as a biomarker to predict the severity of the disease. Host genetic factors or mutations may have been favored by a survival benefit, and a rapid, enhanced release of MC proteases more effectively degrades and/or neutralizes potentially lethal insect venoms or toxins. Indeed, a patient with anaphylaxis to Hymenoptera hosted a mutation in *KARS*, a gene that encodes Lysyl–tRNA synthetase, increasing microphthalmia-associated transcription factor (MITF) activity. MITF is directly involved in histamine and PGD2 production. The Lysyl–tRNA–MITF pathway and its relevance for MC activity were first discovered by Razin et al. In this Special Issue, Govindaraj [9] from Razin’s group reviews the pLysRS–Ap_4_A signaling pathway, providing an additional important step toward a full understanding of the intracellular mechanisms involved in MC activation and beyond. In this context, to further fathom the MC signals that may promote adverse effects, in this Special Issue, Quan et al. [10] review the latest data about the hot and emerging topic of MRGPRX2 receptor. Natural ligands of MRGPRX2 include host defense peptides, basic molecules, and key neuropeptides, such as substance P and vasoactive intestinal peptides. On the other hand, MRGPRX2 has been linked to the pathophysiology of non-IgE-mediated immediate hypersensitivity drug reactions. Its role extends beyond allergic diseases such as asthma, atopic dermatitis, contact dermatitis, and chronic spontaneous urticaria. The independent IgE mechanism of MC activation deserves attention; therefore, in this Special Issue, Voss et al. [11] discuss the protective role of MCs in host defense maintaining skin integrity or sustaining a chronic skin inflammation, emphasizing the importance of IgE-independent pathways of MC activation in connective tissue. Among other tissues, MCs are found in the testis or the epididymis in most mammals, as reviewed in this Special Issue by Himelreich-Perić et al. [12]. The response of several pathological conditions of the mammalian male reproductive system has also been related to MC activation status.

Altogether, the data presented in this issue agree with the dual role of MCs in host defense and pathology. MCs are programmed and located to be sentinels in the body; they are not just the cells that first encounter pathogens, but they also deliver very early responses that result in changes in the local environment and circulating immune cells. It becomes more evident that an increased ability of MCs to function effectively in host defense, perhaps promoted by the benefits of survival, may convert an inappropriate activation into an undesirable pathological state linked to chronic inflammatory diseases or anaphylaxis (Figure 1). Enhanced knowledge regarding how to address inappropriate MC activation will be key to understanding and treating MC-based diseases.

## Figures and Tables

**Figure 1:**
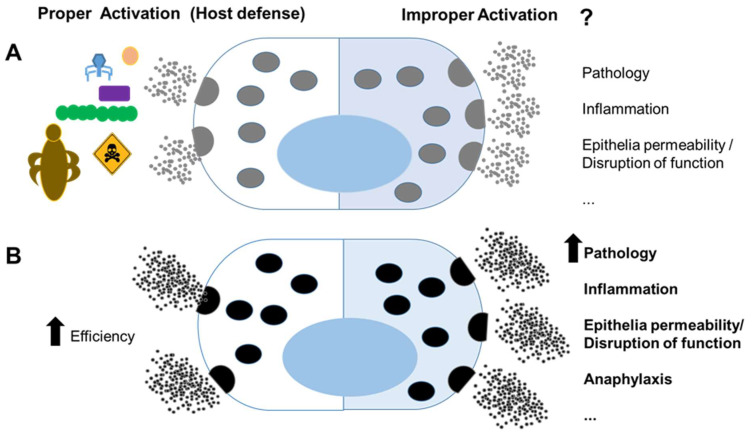
Mast cells in host defense and pathology: (**A**) mast cells have recognized roles in immunity against pathogens and venoms (**left**); however, improper activation against innocuous agents or dysregulated activation may lead to pathology (**right**); (**B**) mast cells that host genetic factors and mutations, possibly promoted by benefits of survival, more efficiently clear pathogen and venoms (**left**); however, they can lead to a more severe pathology under inappropriate activation (**right**).
